# Relationship between blood eosinophil levels and COVID-19 mortality^☆^

**DOI:** 10.1016/j.waojou.2021.100521

**Published:** 2021-02-11

**Authors:** Bingdi Yan, Junling Yang, Yan Xie, Xiaolei Tang

**Affiliations:** a Department of Respiratory and Critical Care Medicine, The Second Hospital of Jilin University, Changchun, Jilin, PR China; b Office of Institutional Research, University of Redlands, Redlands, CA, USA; c Department of Veterinary Biomedical Sciences, College of Veterinary Medicine, Long Island University, Brookville, NY, USA; d Division of Regenerative Medicine, Department of Medicine, Department of Basic Sciences, School of Medicine, Loma Linda University, Loma Linda, CA, USA

**Keywords:** SARS-CoV-2, COVID-19, Eosinophils, Eosinopenia, Mortality

## Abstract

**Objectives:**

A novel coronavirus, Severe Acute Respiratory Syndrome Coronavirus 2 (SARS-CoV-2), is causing the worldwide coronavirus disease 2019 (COVID-19) outbreak with high mortality. A unique finding among COVID-19 patients was a decline of eosinophil levels (eosinopenia). However, results from previous studies on the relationship between eosinopenia and disease severity were inconsistent. The objective of this study is to determine the relationship between eosinopenia and COVID-19 mortality as well as the clinical conditions that could potentially lead to mortality.

**Methods:**

One hundred ninety patients diagnosed as moderate, severe, or critical COVID-19 at hospital admission were enrolled. Data collected from patients’ medical records on the second day after hospital admission included medical histories, clinical symptoms, chest images of computed tomography (CT), laboratory examinations, and outcomes.

**Results:**

Eosinophil levels were significantly lower in patients with critical disease, when compared to those with moderate and severe diseases. After controlled for confounding factors, ie, age, gender, hypertension, coronary heart disease, diabetes, and chronic lung disease, a progressive decline of eosinophil levels was independently associated with mortality. Moreover, eosinophil levels significantly and positively correlated with platelet and D-dimer levels but significantly and inversely correlated with serum levels of urea, creatinine, aspartate aminotransferase, lactate dehydrogenase, and creatine kinase.

**Conclusions:**

Eosinopenia, if progressively worsening, indicates that COVID-19 patients may progress to critical disease and have a significantly higher chance of mortality. Additionally, eosinopenia correlates with biomarkers of coagulation disorder and those of tissue damage in kidney, liver, and other tissues.

## Introduction

Coronaviruses are enveloped viruses. Spike glycoproteins (S protein), envelope proteins (E protein), and membrane glycoproteins (M protein) are attached to the envelope. Inside the envelope, nucleocapsid proteins (N protein) are wrapped with a single-stranded, non-segmented, positive-sense RNA. The sizes of the RNA genome range from 26 to 32 kilobases.^1^ Coronaviruses are common causes of human diseases. Some of them mainly infect upper respiratory tract and cause mild symptoms. These human coronaviruses include 229E, NL63, OC43, and HKU1. Whereas, there are 3 coronaviruses that commonly infect the lower respiratory tract and the diseases caused by these coronaviruses can be fatal. These 3 viruses are Severe Acute Respiratory Syndrome Coronavirus (SARS-CoV), Middle East Respiratory Syndrome Coronavirus (MERS-CoV), and Severe Acute Respiratory Syndrome Coronavirus 2 (SARS-CoV-2).^2^

The SARS-CoV-2 is currently causing the worldwide outbreak of infection (COVID-19) that occurred in Wuhan, China in December 2019.^2^^,^^3^ COVID-19 patients at disease onset typically presented with fever, nonproductive cough, and myalgia/fatigue. Some patients developed dyspnea, pneumonia, acute respiratory distress syndrome, acute cardiac injury, secondary infection, and multiple organ failure. Mortality rate could be up to 21%.^4^^,^^5^ With respect to laboratory findings, for the patients with severe disease, in addition to increased levels of inflammatory markers such as C-reactive protein, D-dimer, and procalcitonin, some other typical findings included an increase of neutrophil counts (neutrophilia)^5^ and a decline of lymphocyte levels (lymphopenia).^5^^,^^6^ The above laboratory findings were shared with SARS-CoV and MERS-CoV.^7^^,^^8^

A unique finding associated with SARS-CoV-2 infection was a decline of eosinophil levels (eosinopenia).^9^, ^10^, ^11^, ^12^, ^13^, ^14^ However, results from previous studies on the relationship between eosinopenia and disease severity were inconsistent.^12^, ^13^, ^14^ Because COVID-19 can lead to high mortality, this study aimed to determine the relationship between eosinopenia and COVID-19 mortality as well as the clinical conditions that could potentially lead to mortality by retrospectively analyzing 190 COVID-19 patients with different disease severities, ie, moderate, severe, or critical.

## Methods

### Patients

This study enrolled patients who were admitted between January 28 and March 25, 2020. The definitive cases of COVID-19 were diagnosed by positive RT-qPCR results in pharyngeal swab specimens.

### Clinical classifications

According to the Guidelines for the Diagnosis and Treatment of COVID-19 (5th version), disease severities were classified as mild, moderate, severe, and critical. In this study, we enrolled patients with moderate, severe, and critical cases, which were judged at the time of admission. Moderate cases: Patients had general respiratory infection symptoms such as fever and nonproductive cough. In addition, patients displayed symptoms of lower respiratory tract infection such as dyspnea as well as pneumonia manifestations in chest images. Severe cases: All severe cases met all the following conditions — Respiratory rate ≥30 per min; Oxygen saturation ≤93% at rest; arterial blood oxygen partial pressure (PaO2)/fraction of inspired oxygen (FiO2) ≤300 mmHg. In addition, patients displayed greater than 50% progression of pulmonary lesions in chest images within 24–48 h. Critical cases: All the critical cases met one of the following criteria — respiratory failure occurs and mechanical ventilation is needed; shock; other organ failures requiring intensive care unit monitoring and treatment.

### Data collection

Data were collected from patients’ medical records. The following data were collected: medical histories, clinical symptoms, chest CT images, laboratory examinations, and outcomes. Outcome data were updated on March 25, 2020. Laboratory examinations included a complete blood count (XE-2100 automated Hematology System, Sysmex); biomarkers for coagulation (prothrombin time, fibrinogen, D-dimer, fibrinogen degradation products, and platelet count), kidney function (urea, creatinine, and estimated glomerular filtration rate), liver function (aspartate aminotransferase and alanine aminotransferase), tissue damage (lactate dehydrogenase, creatine kinase, and total amylase), and infection (C-reactive protein, erythrocyte sedimentation rate, procalcitonin, and ferritin). The above data were collected on the second day after admission. Two doctors independently reviewed the data collection forms to ensure accuracy. Characteristics of the enrolled 190 COVID-19 patients are summarized in Table 1.

**Table 1:** Characteristics of enrolled patients

Characteristics	All (n = 190)	Disease Severity		
		Moderate (n = 69)	Severe (n = 80)	Critical (n = 41)
Ages: median (range)	59.5 (14–86)	48 (14–81)	62.5 (28–86)	69 (26–83)
Numbers of patients within different age groups				
<40 yrs	31	21	9	1
40–64 yrs	84	30	38	16
65–74 yrs	52	15	22	15
>74 yrs	23	3	11	9
Numbers of patients within different gender groups				
Male	102	28	44	30
Female	88	41	36	11
Numbers of patients showing the indicated symptoms				
Fever	161	59	63	39
Cough	137	42	63	32
Expectoration	55	16	30	9
Dyspnea	111	28	48	35
Hemoptysis	10	6	3	1
Fatigue	126	37	60	29
Diarrhea	38	16	14	8
Poor appetite	89	23	44	22
Numbers of patients with indicated comorbidities				
Hypertension	58	15	23	20
Cardiovascular disease	21	4	7	10
Diabetes	33	7	16	10
Chronic lung disease	10	4	2	4
Chronic bronchitis	5	3	1	1
Bronchiectasis	1	1		
Asthma	1		1	
Emphysema	1			1
Tuberculosis	1			1
Chronic Obstructive pulmonary disease (COPD)	1			1

### Study approval

The study was performed in accordance with the Declaration of Helsinki principles for ethical research. The study was approved by the Ethics Commission of the Second Hospital of Jilin University.

### Statistical analysis

All statistical analyses were performed using R-3.6.3 and RStudio Version 1.2.5042. Continuous variables were assessed for normality of distribution using the Shapiro-Wilk test, with p-value lower than 0.05 as the criterion that a variable deviates from normality.

Independent nominal variables and continuous variables were compared among the three severity levels (moderate, severe, critical) and between the two mortality outcomes (survivors and non-survivors). Logistic regression with eosinophil levels as independent variable was performed to predict mortality.

Frequency comparisons were made using Chi square test. Continuous variables were compared by using unpaired Wilcoxon Rank Sum Tests. For more than two groups, pairwise p-values were adjusted using the Bonferroni correction method, in which the p-values were multiplied by the number of comparisons. Two-tailed p value of less than 0.05 was considered statistically significant: ∗∗∗∗P < 0.0001, ∗∗∗P < 0.001, ∗∗P < 0.01, ∗P < 0.05, #: Not significant.

Correlation matrix of continuous variables were generated using the nonparametric Spearman correlation, which does not make any assumptions about the underlying distribution.

## Results

### Eosinophil levels were significantly lower in COVID-19 patients with critical disease, when compared to those with moderate and severe diseases

**Fig. 1:**
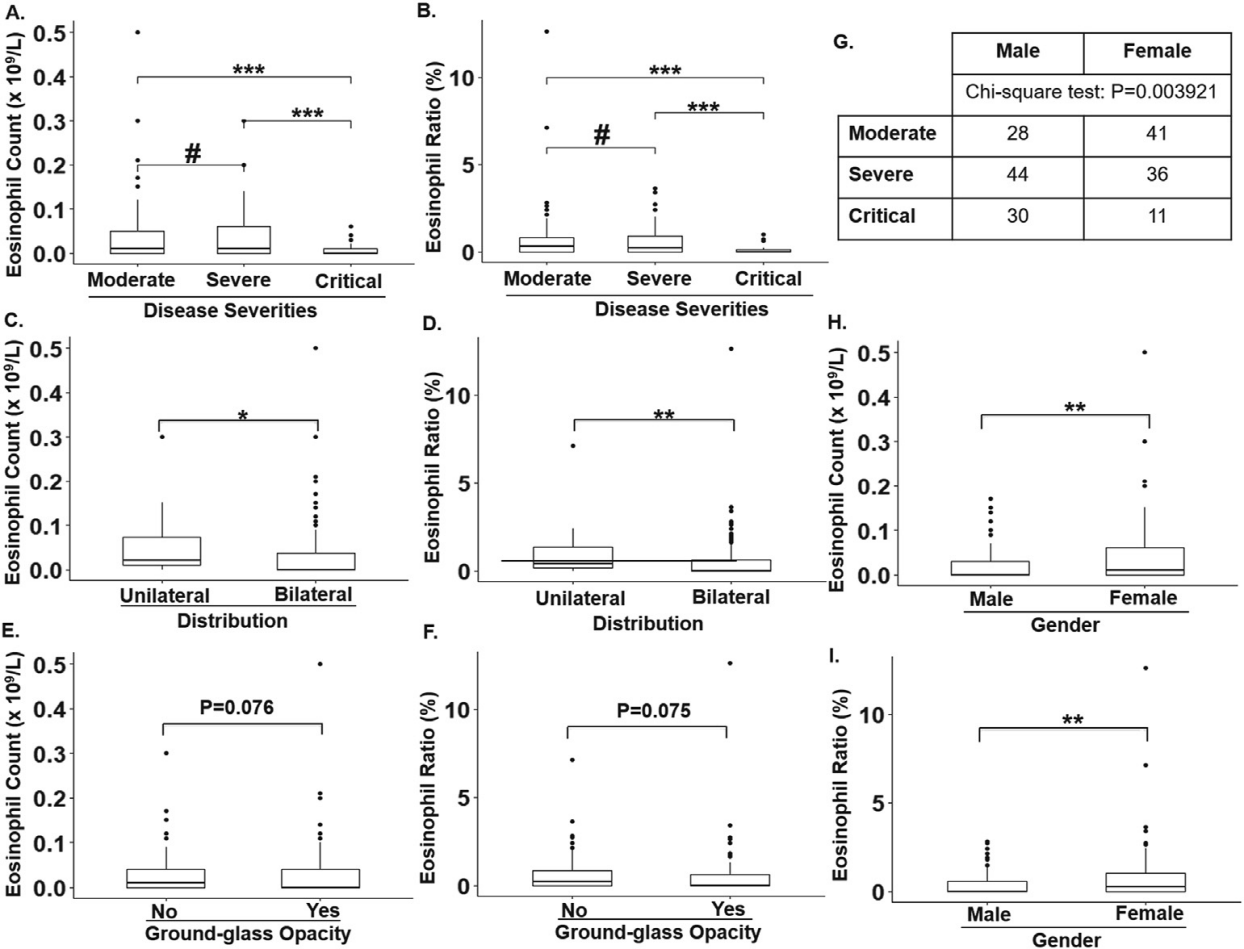
**Eosinophil counts and ratios were significantly lower in COVID-19 patients with critical disease, when compared to those with moderate and severe diseases**. *A)* Eosinophil counts among patients with moderate, severe, and critical diseases. *B)* Eosinophil ratios among patients with moderate, severe, and critical diseases. ∗∗∗P < 0.001; #: Not significant. Kruskal-Wallis test. C*)* Eosinophil counts among patients with unilateral pneumonia and those with bilateral pneumonia. *D)* Eosinophil ratios among patients with unilateral pneumonia and those with bilateral pneumonia. *E)* Eosinophil counts among patients with no ground-glass opacity in chest CT images and those with ground-glass opacity. *F)* Eosinophil ratios among patients with no ground-glass opacity in chest CT images and those with ground-glass opacity. *G)* Numbers of male and female patients who had moderate, severe, and critical diseases. *H)* Eosinophil counts between male and female patients. *I)* Eosinophil ratios between male and female patients. ∗P < 0.05; ∗∗P < 0.01. Wilcoxon test

We first asked whether eosinopenia was associated with disease severity. We did not see a significant difference in eosinophil counts (normal range: 0.02–0.52 × 10^9^/L) and ratios (normal range: 0.4–8%) between patients with moderate disease and those with severe disease (Fig. 1A and B). However, eosinophil counts and ratios were significantly lower in patients with critical disease when compared to those with moderate and severe diseases (Fig. 1A and B).

Because patients with bilateral pneumonia and ground-glass opacity in chest CT images commonly experienced respiratory failure and required mechanical ventilation that were the criteria for the diagnosis of critical disease, we compared the eosinophil counts and ratios in patients with bilateral pneumonia and ground-glass opacity in chest CT images with those with unilateral pneumonia and no ground-glass opacity. Our data showed that the patients with bilateral pneumonia in chest CT images, when compared to those with unilateral pneumonia, had significantly lower eosinophil counts (Fig. 1C) and ratios (Fig. 1D). In addition, the patients with ground-glass opacity in chest CT images, when compared to those with no ground-glass opacity, trended towards lower eosinophil counts (Fig. 1E) and ratios (Fig. 1F), although these changes were not statistically significant.

In addition, we found that significantly more male patients, when compared to female patients, presented with critical disease (Fig. 1G). For this reason, we compared eosinophil counts and ratios in male patients with those in female patients. Consistent with this finding, our data further showed that male patients, when compared to female patients, had significantly lower levels of eosinophil counts (Fig. 1H) and ratios (Fig. 1I).

### A progressive decline of eosinophil levels, after controlled for confounding factors, was associated with mortality among COVID-19 patients

**Fig. 2:**
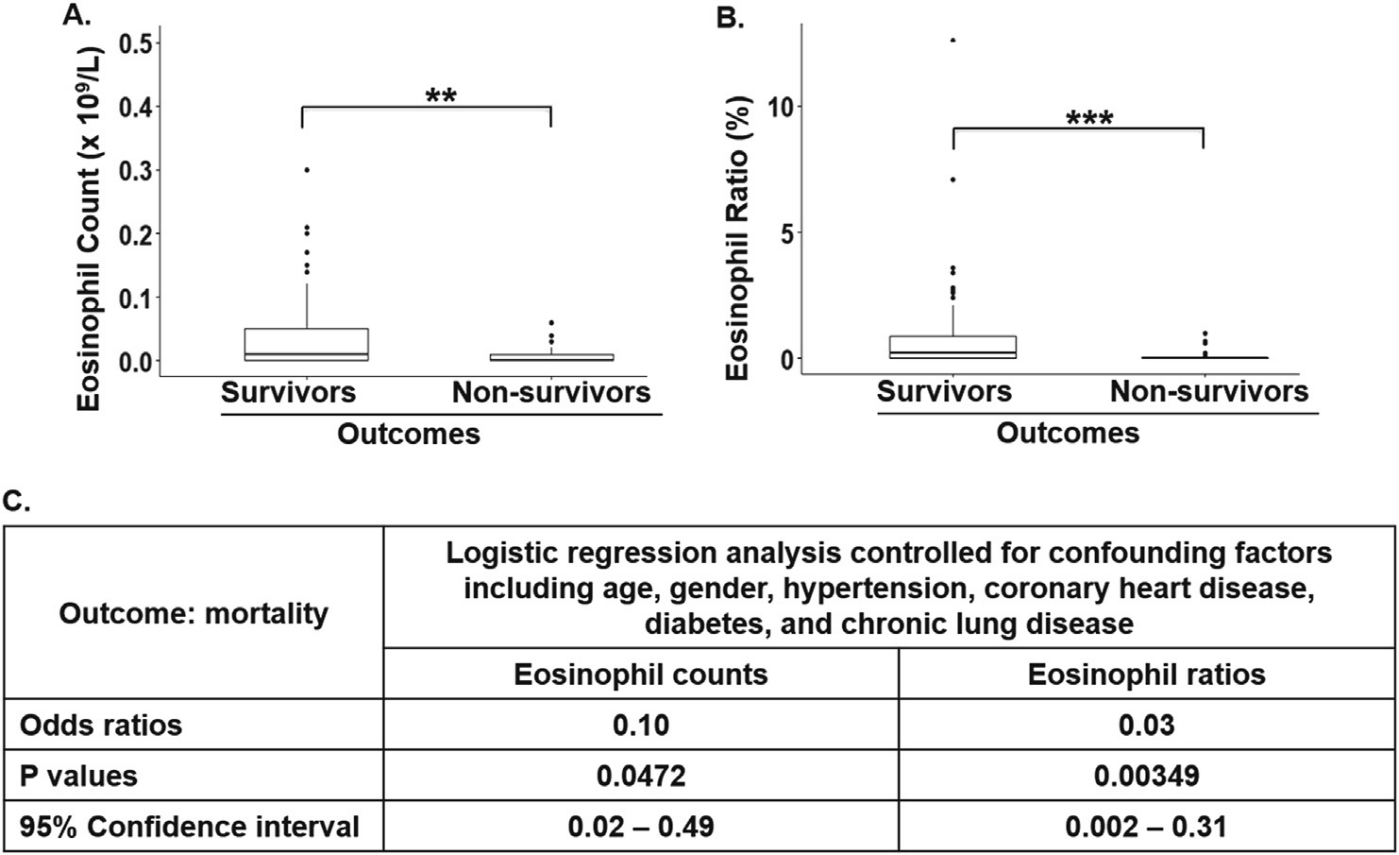
**A progressive decline of eosinophil counts and ratios, after controlled for confounding factors, was associated with mortality among COVID-19 patients. *A)* A comparison of eosinophil counts between survivors and non-survivors**. *B)* A comparison of eosinophil ratios between survivors and non-survivors. ∗∗P < 0.01; ∗∗∗P < 0.001. Wilcoxon test. *C)* Regression analysis to determine whether a progressive decline of eosinophil counts and ratios, after controlled for confounding factors, was associated with mortality among COVID-19 patients

The finding that eosinophil counts and ratios were severely suppressed in COVID-19 patients with critical disease prompted us to ask whether eosinophil counts and ratios were associated with mortality during SARS-CoV-2 infection. We first compared eosinophil counts and ratios among survivors and non-survivors. Our data showed that non-survivors, when compared to survivors, had significantly lower levels of eosinophil counts (Fig. 2A) and ratios (Fig. 2B).

Based on the above findings, we further asked whether eosinophil counts and ratios were associated with mortality among COVID-19 patients. Our analysis showed that, after controlled for confounding factors including age, gender, hypertension, coronary heart disease, diabetes, and chronic lung disease, the odds ratio was 0.10 (95% CI = 0.02–0.49; p = 0.0472) for eosinophil counts and 0.03 (95% CI = 0.002–0.31; p = 0.00349) for eosinophil ratios (Fig. 2C). Based on these data, it was estimated that, with a decrease of one standard deviation in eosinophil counts or ratios, there was an increase of approximately 90% or 97% odds in mortality respectively.

### Eosinophil levels in COVID-19 patients significantly and positively correlated with biomarkers of coagulation disorder

COVID-19 mortality may be due to multiple factors such as coagulation disorder^15^ and multiple organ failure.^16^ Hence, we asked whether eosinophil levels were associated with biomarkers of coagulation disorder and tissue damage.

Firstly, we found that eosinophil counts and ratios significantly and inversely correlated with infection markers (Fig. S1), ie, C-reactive protein (CRP), procalcitonin (PRO), and ferritin (FER). The data suggest that progressive decline of eosinophil counts and ratios indicates worsening of viral infection.

**Fig. 3:**
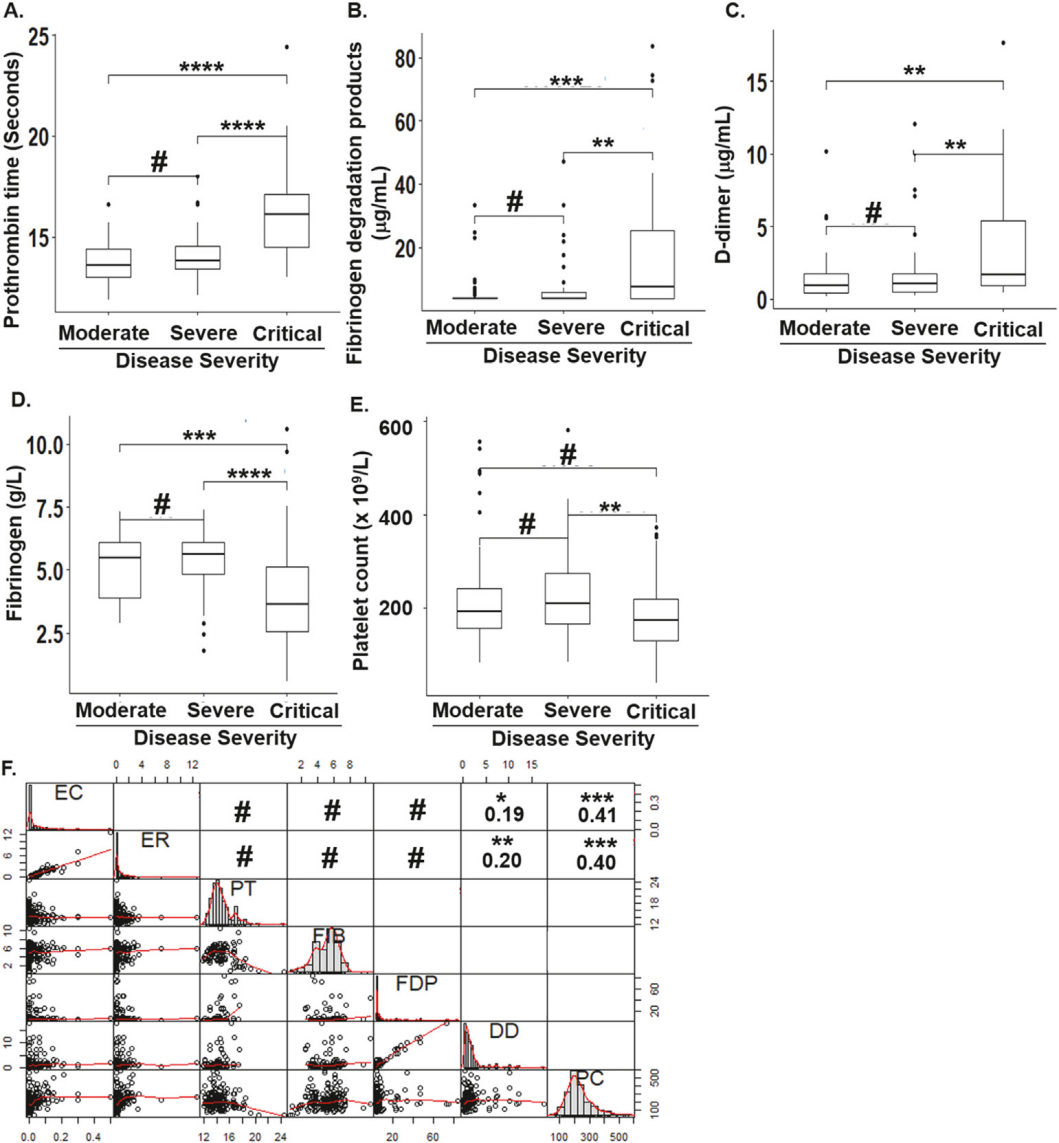
**Eosinophil counts and ratios in COVID-19 patients correlated with biomarkers of coagulation disorder**. *A)* Prothrombin time. *B)* Fibrinogen degradation products. *C)* D-dimer. *D)* Fibrinogen. *E)* Platelet count. ∗∗P < 0.01; ∗∗∗P < 0.001; ∗∗∗∗P < 0.0001; #: not significant. Kruskal-Wallis test. *F)* Correlation among eosinophil count (EC), eosinophil ratio (ER), prothrombin time (PT), fibrinogen (FIB), fibrinogen degradation products (FDP), D-dimer (DD), and platelet count (PC). Lower left corner shows correlation dot plots. Upper right corner shows R and P values. Numbers inside the squares are “R” values. ∗P < 0.05; ∗∗P < 0.01; ∗∗∗P < 0.001; #: not significant. Spearman Correlation analysis

With respect to coagulation disorders, our data showed no significant difference between patients with severe disease and those with moderate disease in the levels of coagulation biomarkers, ie, prothrombin time (Fig. 3A), fibrinogen degradation products (Fig 3B), D-dimer (Fig. 3C), fibrinogen (Fig. 3D), and platelet count (Fig. 3E). However, we found that patients with critical disease, when compared to those with severe diseases, showed significantly increased levels of prothrombin time (Fig. 3A), fibrinogen degradation products (Fig 3B), and D-dimer (Fig. 3C). In contrast, patients with critical disease, when compared to those with severe disease displayed significantly decreased levels of fibrinogen (Fig. 3D) and platelet count (Fig. 3E).

The above observations prompted us to investigate the potential correlation of eosinophil counts and ratios with the above coagulation biomarkers. Our data showed that eosinophil counts and ratios significantly and positively correlated with platelet counts (Fig. 3F), which was consistent with previous reports that eosinophils and platelets interacted with each other.^17^ In addition, eosinophil counts and ratios also significantly and positively correlated with D-dimer levels (Fig. 3F).

### Eosinophil levels in COVID-19 patients significantly and inversely correlated with biomarkers of kidney injury

**Fig. 4:**
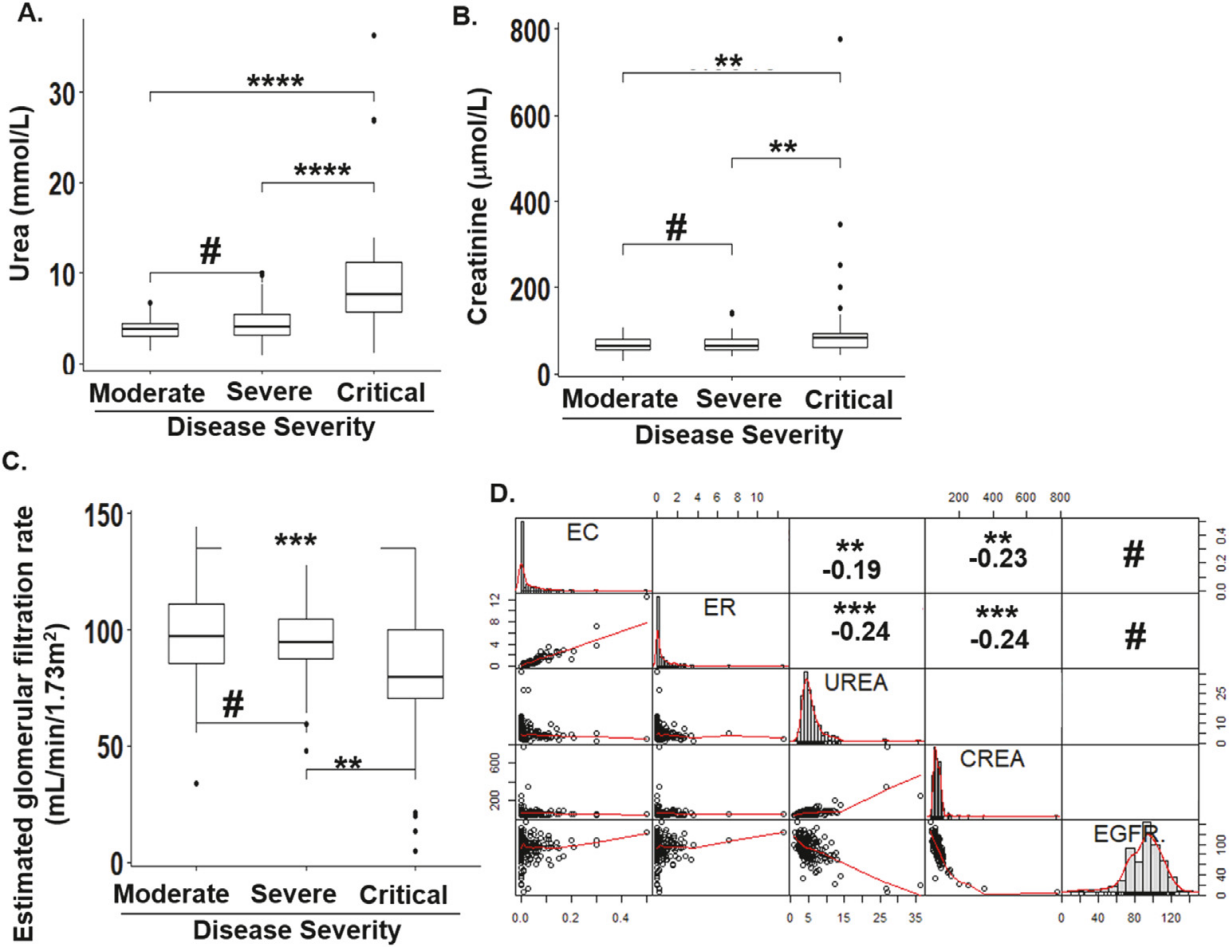
**Eosinophil counts and ratios in COVID-19 patients significantly and inversely correlated with biomarkers of kidney injury**. *A)* Serum urea levels. *B)* Serum creatinine levels. *C)* Estimated glomerular filtration rates. ∗∗P < 0.01; ∗∗∗P < 0.001; ∗∗∗∗P < 0.0001; #: none significant. Kruskal-Wallis test. *D)* Correlation analysis among eosinophil count (EC), eosinophil ratios (ER), urea, creatinine (CREA), and estimated glomerular filtration rates (EGFR). Lower left corner shows correlation dot plots. Upper right corner shows R and P values. Numbers inside the squares are “R” values. ∗∗P < 0.01; ∗∗∗P < 0.001; #: not significant

Next, we asked whether eosinopenia was associated with biomarkers of kidney injury. Our data showed that between patients with severe disease and those with moderate disease, there were no significant differences in the serum levels of urea (Fig. 4A) and creatinine (Fig. 4B) as well as estimated glomerular filtration rates (Fig. 4C). Whereas, our analyses revealed that the patients with critical disease, when compared to those with moderate and severe diseases, displayed significantly increased serum levels of urea (Fig. 4A) and creatinine (Fig. 4B) but decreased estimated glomerular filtration rate (Fig. 4C), indicating kidney injury. Correlation analysis demonstrated that eosinophil counts and ratios significantly and inversely correlated with serum levels of urea and creatinine (Fig. 4D).

### Eosinophil levels in COVID-19 patients significantly and inversely correlated with biomarkers of liver injury

**Fig. 5:**
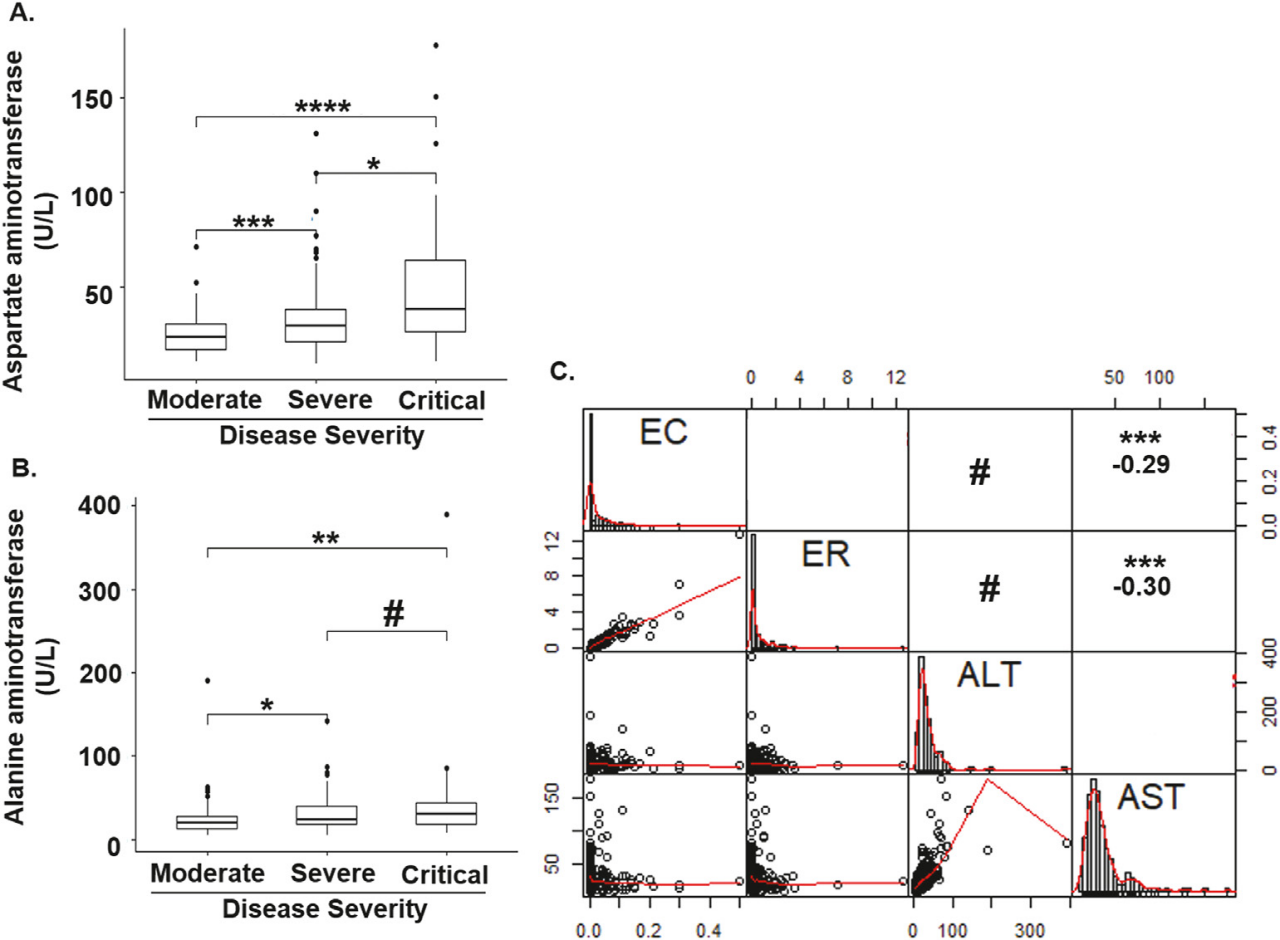
**Eosinophil counts and ratios in COVID-19 patients significantly and inversely correlated with biomarkers of liver injury**. *A)* Serum levels of aspartate aminotransferase. *B)* Serum levels of alanine aminotransferase. ∗P < 0.05; ∗∗P < 0.01; ∗∗∗P < 0.001; ∗∗∗∗P < 0.0001. Kruskal-Wallis test. *C)* Correlation analysis among eosinophil count (EC), eosinophil ratio (ER), alanine aminotransferase (ALT), and aspartate aminotransferase (AST). Lower left corner shows correlation dot plots. Upper right corner shows R and P values. Numbers inside the squares are “R” values. ∗∗∗P < 0.001

We then investigated the effects of eosinopenia on biomarkers of liver injury. We found that patients with severe disease, when compared to those with moderate disease, showed significantly increased serum levels of aspartate aminotransferase (Fig. 5A) and alanine aminotransferase (Fig. 5B), indicating liver injury. In addition, patients with critical disease displayed further significantly increased serum levels of aspartate aminotransferase (Fig. 5A) but not those of alanine aminotransferase (Fig. 5B), suggesting worsening of liver injury in patients with critical disease. Correlation analyses demonstrated that eosinophil counts and ratios significantly and inversely correlated with serum levels of aspartate aminotransferase (Fig. 5C).

### Eosinophil levels in COVID-19 patients significantly and inversely correlated with biomarkers of tissue damage

**Fig. 6:**
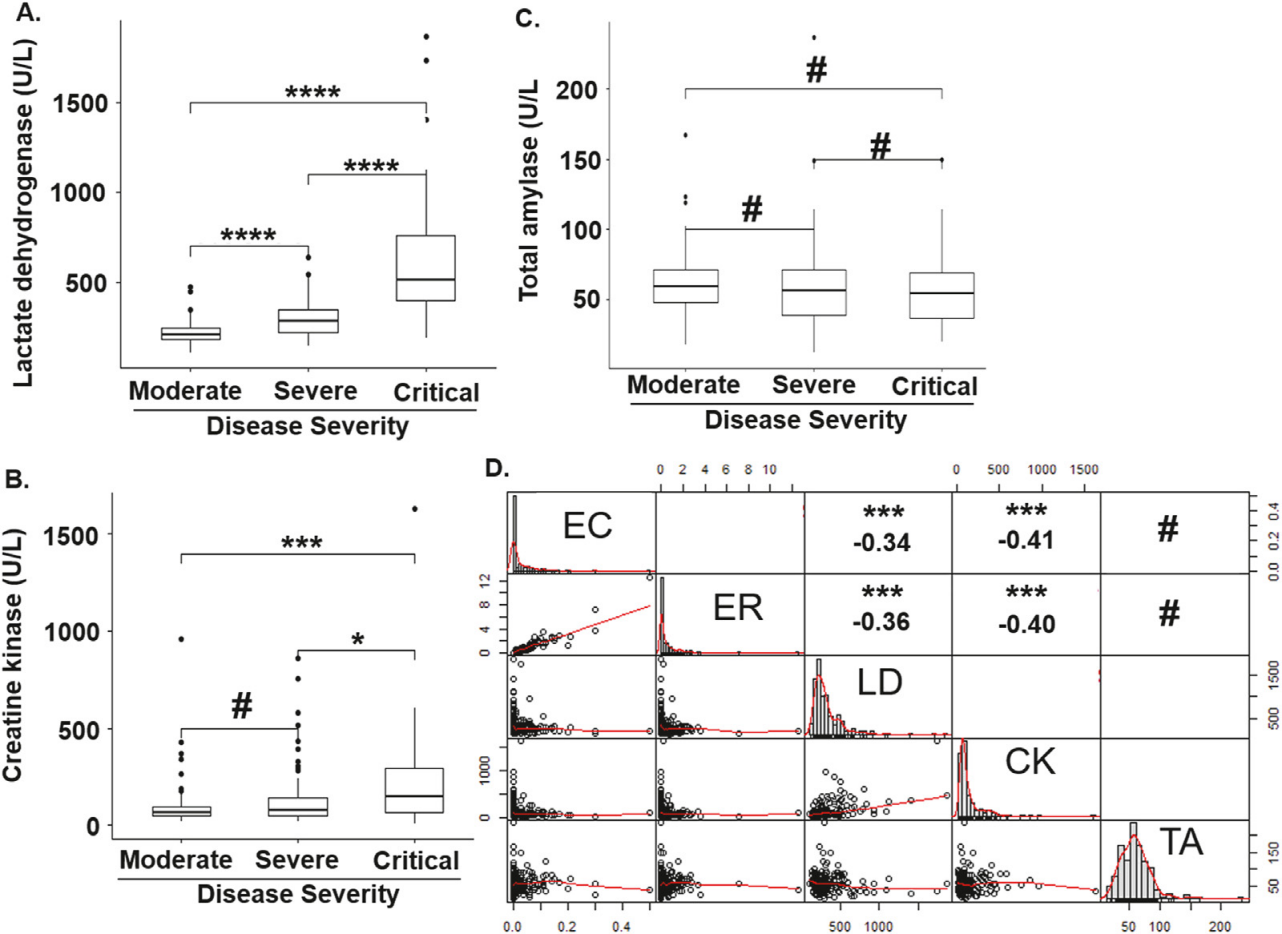
**Eosinophil counts and ratios in COVID-19 patients significantly and inversely correlated with biomarkers of tissue damage**. *A)* Serum levels of lactate dehydrogenase. *B)* Serum levels of creatine kinase. C) Serum levels of total amylase. ∗P < 0.05; ∗∗∗P < 0.001; ∗∗∗∗P < 0.0001, #: not significant. Kruskal-Wallis test. *C)* Correlation analysis among eosinophil count (EC), eosinophil ratio (ER), lactate dehydrogenase (LD), creatine kinase (CK), and total amylase (TA). Lower left corner shows correlation dot plots. Upper right corner shows R and P values. Numbers inside the squares are “R” values. ∗∗∗P < 0.001

Finally, we investigated the effects of eosinopenia on other biomarkers of tissue damage. Our analyses showed that patients with severe disease, when compared to those with moderate disease, had significantly increased serum levels of lactate dehydrogenase (Fig. 6A) but not creatine kinase (Fig. 6B), suggesting tissue damage. In addition, patients with critical disease, when compared to those with moderate and severe diseases, displayed significantly further increase in serum levels of both lactate dehydrogenase (Fig. 6A) and creatine kinase (Fig. 6B), suggesting worsening of tissue damage. We did not see significant differences in the serum levels of total amylase among the three disease severities (Fig. 6C).

Correlation analyses demonstrated that eosinophil counts and ratios significantly and inversely correlated with the serum levels of lactate dehydrogenase and creatine kinase, but not total amylase (Fig. 6D).

## Discussion

In this study, we demonstrated that eosinophil levels were significantly lower in COVID-19 patients with critical disease, when compared to those with moderate and severe diseases. Importantly, we showed that a progressive decline of eosinophil levels, after controlled for confounding factors, was associated with mortality among COVID-19 patients. In addition, our analyses revealed that eosinopenia correlated with biomarkers of coagulation disorder and those of tissue damage in kidney, liver, and other tissues. Therefore, our data suggest that eosinopenia can be a potential target in the treatment of COVID-19.

The well-established functions of eosinophils include their association with parasitic infection and asthma. Under these conditions, eosinophil numbers are increased in blood (eosinophilia). Previous studies suggest that eosinophils protect against parasitic infections but play a damaging role in the case of severe asthma. However, recently emerging data from mouse studies show that eosinophils have anti-viral activity.^18^^,^^19^ These animal data were further corroborated by other studies, in which a humanized anti-IL-5 monoclonal antibody (Mepolizumab), which effectively treated eosinophilic asthma by reducing eosinophil numbers, increased viral loads in both humans and mice.^20^^,^^21^ However, an intriguing finding was that eosinophils particularly those from severe asthma patients had a reduced ability to capture viruses.^22^ The above findings suggest, during virus-induced asthma exacerbations, that preservation of physiological eosinophil counts and anti-viral function can be important for fighting virus infections.

At least 2 biological functions may contribute to the eosinophil's anti-viral immunity. Firstly, eosinophils, when being activated by viruses, release molecules such as neurotoxin/ribonuclease 2 and cationic proteins that can kill viruses.^18^ Secondly, eosinophils express molecules such as toll-like receptors, CD80, CD86, and major histocompatibility complex I/II molecules that are sufficient for stimulating an immune response. To support this notion, it was shown that influenza A virus-infected eosinophils can act as professional antigen-presenting cells and stimulate virus-specific CD8^+^ T cell-mediated antiviral immune response in vivo.^19^ This is important because, if eosinophils play a role in the immune defense against SARS-CoV-2, strategies to correct the eosinopenia in COVID-19 patients are justified to prevent fatality.

Moreover, our studies demonstrated that a progressive decline of eosinophil levels was associated with mortality among COVID-19 patients (Fig. 2). This finding was also corroborated by other findings in this current study. Firstly, consistent with other reports,^15^ we found that COVID-19 patients with critical disease had coagulation disorders (Fig. 3). In addition, our analysis revealed a significantly positive correlation of eosinophil counts and ratios with platelet counts (Fig. 3). This finding can be potentially important because previous studies have shown that eosinophils and platelets interact with each other^17^ and that eosinophil counts inversely correlated with stroke severity.^23^ Secondly, we found that COVID-19 patients with critical disease showed biomarkers of tissue injury (Fig. 4, Fig. 5, Fig. 6). Importantly, eosinophil counts and ratios significantly and inversely correlated with some of these biomarkers, suggesting that eosinopenia was associated with the organ failure and tissue damage. Hence, our data support the emerging concept that eosinophils promote tissue repair.^24^, ^25^, ^26^ For this reason, we propose to analyze eosinophils in both blood and tissues in details.

The strength of our study is that we, for the first time, systemically analyzed the relationship between eosinopenia severity and COVID-19 disease severity. We reason that such a detailed, focused analysis of eosinophils in the context of COVID-19 is necessary because new research data have clearly suggested the importance of eosinophils in the immune defense against virus infections.^18^, ^19^, ^20^

There are several limitations in this study. First, the study population included patients only from a single hospital. Second, this cohort contained only 190 patients, which might not be sufficiently powered for all the analyses. Third, because laboratory results from the only one-time point were available, we could not perform longitudinal studies. Fourth, to be consistent with other white blood cell counts, the eosinophil count unit used in current clinical practice is x10^9^/L. This unit is okay when the allergy is analyzed, in which eosinophil count is dramatically increased. However, it will be challenging to visualize eosinopenia using this current unit because of the relatively low eosinophil count. For this reason, we suggest that the unit of eosinophil count should be adjusted to x10^7^ ~ 10^8^/L, which allows easier visualization of both eosinophilia and eosinopenia.

## Abbreviations

ALT: Alanine aminotransferase; AST: Aspartate aminotransferase; COVID-19: Coronavirus disease 2019; CK: Creatine kinase; CT: Computed tomography; DD: D-dimer; EC: Eosinophil count; ER: Eosinophil ratio; EGFR: Estimated glomerular filtration rates; FIB: Fibrinogen; FDP: Fibrinogen degradation products; FiO2: Fraction of inspired oxygen; LD: Lactate dehydrogenase; MERS-CoV: Middle east respiratory syndrome coronarivurs; SARS-CoV: Severe acute respiratory syndrome coronavirus; SARS-Cov-2: Severe acute respiratory syndrome coronavirus 2; PaO2: Arterial blood oxygen partial pressure; PC: Platelet count; PT: Prothrombin time; RT-qPCR: Real time quantitative polychain reaction; CREA: Urea creatinine.

## Funding

None.

## Informed consent

The human data included in this study do not contain identifiable personal information. Therefore, informed consent is not applicable.

## Consent for publication

All authors consent to publication of the work in the World Allergy Organization Journal.

## Authorship contributions

XT conceived the research question. BY and JY collected and organized the data. YX performed the statistical analyses. XT and BY analyzed the data and drafted the initial manuscript. All authors critically reviewed the manuscript and participated in writing the final version of this manuscript.

## Availability of data and materials

The de-identified data that support the findings of this study are available upon reasonable request.

## Ethics approval

The study was approved by the Ethics Commission of the Second Hospital of Jilin University.

## Declaration of competing interest

The authors declare no competing interests.
