# Supplementary material for: Relationship between blood eosinophil levels and COVID-19 mortality
Source: World Allergy Organ J. 2021 Feb 11;14(3):100521. doi: 10.1016/j.waojou.2021.100521 (PMC7877210; doi:10.1016/j.waojou.2021.100521)
Supplement: Multimedia component 1 [file mmc1.docx]

**SUPPLEMENTAL DATA**


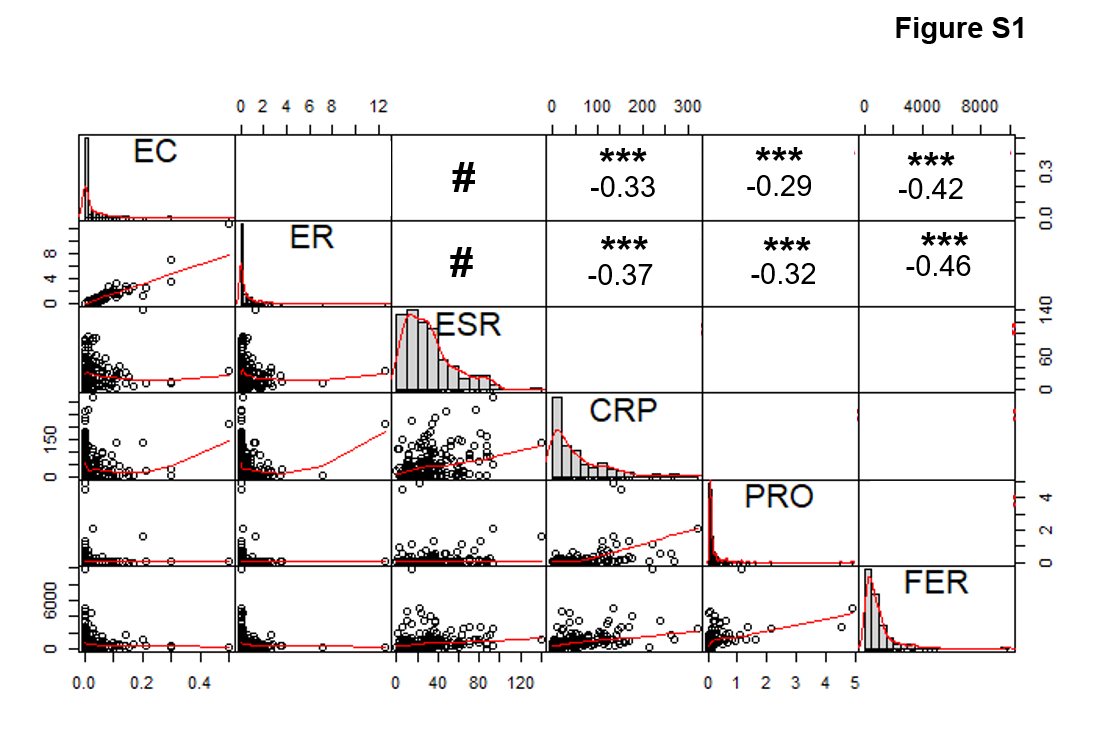
**Figure S1. Eosinophil counts and ratios significantly and inversely correlated with infection markers.** Correlation among eosinophil count (EC), eosinophil ratio (ER), erythrocyte sedimentation rate (ESR), C-reactive protein (CRP), procalcitonin (PRO), and ferritin (FER). Lower left corner shows correlation dot plots. Upper right corner shows R and P values. Numbers inside the squares are “R” values. ***P<0.001; #: not significant. Spearman Correlation analysis.
